# Hypertriglyceridemia Screening in Acute Pancreatitis: Diagnostic Blind Spot in Clinical Routine

**DOI:** 10.1007/s10620-025-09366-4

**Published:** 2025-09-13

**Authors:** Mohamad Amer Nashtar, Jan Kempener, Uttban Gohman, Jasmin Weninger, Eda Kaya, Obayda Azizy, Paul Manka, Mustafa Özcürümez, Polykarpos Christos Patsalis, Ali Canbay, Martin Steinmetz

**Affiliations:** 1 Section of Cardiology, Angiology and Internal Emergency Medicine, Department of Internal Medicine, Knappschaft Kliniken-University Hospital Bochum, Ruhr University Bochum, Bochum, Germany; 2 Section of Laboratory Medicine, Department of Internal Medicine, Knappschaft Kliniken-University Hospital Bochum, Ruhr University Bochum, Bochum, Germany; 3 Section of Hepatology and Gastroenterology, Department of Internal Medicine, Knappschaft Kliniken-University Hospital Bochum, Ruhr University Bochum, Bochum, Germany

**Keywords:** Hypertriglyceridemia, Acute pancreatitis, Acute abdominal pain, Underestimation

## Abstract

**Background:**

Hypertriglyceridemia (HTG) is the third most common cause of acute pancreatitis (AP) after gallstones and alcohol abuse. Early determination of the triglyceride (TG) level in patients with acute abdominal pain, particularly in cases of AP, is crucial, as an acute increase can be followed by a rapid decrease within 2 days. HTG as a cause of acute abdominal pain and AP is often overlooked in clinical practice. In addition, manifest HTG is often underdiagnosed and inadequately treated.

**Methods:**

We investigated 1279 cases of acute abdominal pain, including 226 cases of AP, to assess the frequency and timing of TG level measurement. Additionally, we studied 237 patients with HTG levels above 500 mg/dL to determine the frequency of HTG-related symptoms and the rate of initiation of adequate therapy, considering the specialty in which the elevated TG levels were identified.

**Results:**

Triglycerides were determined in 22% of patients with acute abdominal pain and 55% with AP; fewer than 15% of all patients received a determination at the first contact with the physician. TG levels were measured at a median of 24 h (1–54) in patients with acute abdominal pain and 48 h (24–95) in those with AP after admission. Less than half of patients with HTG received TG-lowering therapy.

Only 5 of 226 cases with AP was identified as HTG-induced, while 13.5% of all cases with HTG above 500 mg/dL had a history of AP.

**Conclusions:**

Our findings support the assumption that HTG and its complications are often underestimated in clinical practice and require more attention. Furthermore, early initiation of appropriate therapy is crucial.

**Supplementary Information:**

The online version of this article (10.1007/s10620-025-09366-4) contains supplementary material, which is available to authorized users.

## Introduction

Over the last 16 years, the number of cases of moderate or severe acute pancreatitis (AP) admitted to hospital has increased, as has the proportion of cases of acute pancreatitis associated with hypertriglyceridemia (HTG–AP). Gallstones and chronic alcohol abuse remain the most common causes of AP, with metabolic diseases, particularly primary or secondary hypertriglyceridemia, being the third most common cause [1]. The prevalence of pancreatitis caused by HTG was reported to be up to 22% [2]. In cases of AP, early detection of HTG is very important to initiate an appropriate therapy and avoid recurrence. HTG is defined as a fasting triglyceride (TG) concentration above 150 mg/dL (1.7 mmol/L). HTG affects about 15–20% of the adult population [3]. Although there is no defined threshold above which HTG triggers AP, the risk steadily increases as TG levels rise [4]. The main risk factors for AP caused by HTG are younger age, male gender, obesity, poorly controlled diabetes, alcoholism, pregnancy, previous pancreatitis, and a personal or family history of hyperlipidemia compared to people without HTG [5, 6]. HTG in chylomicronemia syndrome can cause AP and chronic abdominal pain. Early measurement of TG after admission is important in order to rule out HTG as a differential diagnosis, since TG levels can fall by an average of 69.8% within 48 h, even without any intervention [7]. This study had two complementary aims. Firstly, we aimed to evaluate the frequency and timing of TG level measurements in patients presenting with acute abdominal pain, with a particular focus on those subsequently diagnosed with AP. Secondly, we aimed to assess the clinical management of patients with severe HTG. Through this dual approach, we aimed to identify potential gaps in the recognition and treatment of HTG and its complications, with the ultimate goal of improving diagnostic and therapeutic strategies in acute and chronic care settings.

## Methods

### Patient Cohort

The retrospective analysis was performed in a tertiary care center (Knappschaft Kliniken, University Hospital Bochum, Germany). Patient files of 1279 cases of abdominal pain, including 226 patients with AP, and files of 237 patients with HTG above 500 mg/dL between 2019 and 2024 were obtained and analyzed.

### Patient Selection

The inclusion criteria were selected according to ICD10 classification system for AP or abdominal pain (R10, K85). All patients who presented to the hospital or emergency department with the above-mentioned diagnoses were included in the study. For a second analysis, patients with a TG level above 500 mg/dL were included, regardless of clinical symptoms or reason for the admission. Patient files that did not meet the inclusion criteria were rejected for further analysis.

### Statistical Methods

All data were transferred from the hospital information system to Excel. Statistical analyses were performed using GraphPad Prism (version 10.5, GraphPad Software, San Diego, CA, USA). Continuous variables with a normal distribution were expressed as mean ± standard deviation (SD), while non-normally distributed variables were summarized as median (Q1–Q3). Categorical variables were expressed as absolute numbers (*n*) and percentages (%). For comparisons between two groups, either Student’s *t*-test or the Mann–Whitney *U* test was applied, depending on the distribution of the data. Categorical variables were compared using the *χ*^2^ test. A two-sided *p*-value < 0.05 was considered statistically significant.

## Results

### Allocation and Treatment in Various Specialist Departments

Patients presenting with acute abdominal pain were treated in various medical departments, while the cases with AP were examined and treated in the Department of Internal Medicine. Common causes of abdominal pain included pancreatitis, gastritis, cholecystitis, appendicitis, infectious diseases and tumors (Table 1). However, no etiology was identified in a relevant proportion of cases.

**Table 1:** Causes of acute abdominal pain

Cause	*n* (%)
Acute pancreatitis	226 (17.67)
Chronic pancreatitis	23 (1.79)
Type B gastritis	27 (2.11)
Type C gastritis	50 (3.90)
Infectious disease	75 (5.86)
Cholecystitis	60 (4.69)
Appendicitis	55 (4.30)
Tumor (benign/malignant)	57 (4.45)
Others	277 (20.40)
Unclear/not defined	429 (33.54)

### Acute Abdominal Pain and Determination of Triglycerides (TG)

Of the cases involving acute abdominal pain, 22% (232) received a TG determination during the entire treatment period, and only 10.2% (108) received one on admission. Of those with determined TG levels, 26.7% (62) had HTG. The median TG level for patients with acute abdominal pain was 118 mg/dL (81–158). TG levels in patients with acute abdominal pain were determined at a median of 24 h (1–54) after admission to hospital. 10 Out of 62 patients (16%) with HTG who presented with acute abdominal pain received TG-lowering therapy.

### Acute Pancreatitis, Determination of Triglycerides (TG) and HTG–AP

In patients with AP, the detection rate was 55% (125) over the entire treatment period, compared to 12.8% (29) at admission. TG measurements were performed in only 36 out of 66 cases of AP for which no cause was identified (62 cases were unclear and 4 were idiopathic). Of the 226 patients with AP, 49 (21.7%) were diagnosed based on imaging and clinical presentation alone, without elevated lipase or amylase levels, as is often the case in HTG-induced pancreatitis [8]. Of the patients with AP for whom TG levels were determined, 36% (45) had at least mild HTG [the median TG level was 116 mg/dL (83–193)]. The median time point for TG measurement in patients with AP was 48 h (24–95) after admission. 16 out of 45 (35.5%) patients with HTG received lipid-lowering therapy of any kind. In line with known epidemiological data, biliary and ethanol-related causes were the most common triggers of AP. HTG was identified as a cause in five patients (Table 2). Compared to the overall AP patient cohort (*n* = 226), these patients were significantly younger, with a mean age of 41 ± 10 years versus 59 ± 19 years (*p* = 0.0286). Four of the five HTG-AP patients were male and four had a BMI > 30 kg/m^2^. All five patients exhibited hepatic steatosis and four had pre-existing diabetes mellitus. Additionally, all five exhibited mixed hyperlipidemia. Notably, none of the patients with HTG-AP was receiving TG lowering medication before admission.

**Table 2:** Causes of acute pancreatitis

Cause	*n* (%)
Biliary	60 (26.54)
Ethyltoxic	54 (23.89)
Tumor (benign/malignant)	5 (2.21)
Post ERCP	13 (5.75)
Hereditary	8 (3.53)
Hypercalcemia	1 (0.44)
Hypertriglyceridemia	5 (2.21)
Pancreas divisum	5 (2.21)
Drug-induced	6 (2.65)
Autoimmune	1 (0.44)
Infectious disease	2 (0.88)
Ideopathic	4 (1.76)
Unclear/not defined	62 (27.43)

**Fig. 1:**
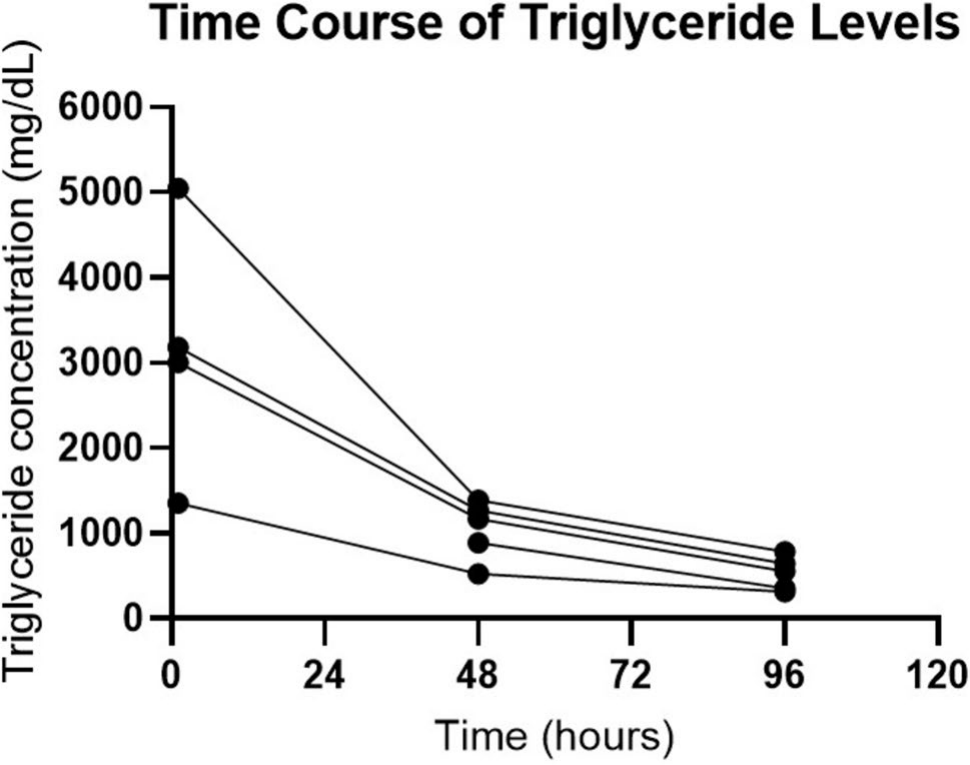
Time course of triglyceride levels in HTG–AP patients over 96 h after admission

The median TG level in patients with HTG–AP was markedly elevated, with a median level of 3002 mg/dL (1118–4113). TG levels were measured at the time of admission in four out of five patients with HTG–AP, while in one case, the measurement was performed 24 h later. TG levels decreased rapidly, with concentrations dropping by more than 50% within the first 48 h of hospitalization (Fig. 1). Interestingly, serum lipase levels in HTG–AP patients tended to be lower than in the overall AP cohort, although this difference was not statistically significant due to the small HTG–AP group sample size. The median lipase level in HTG–AP patients was 101.3 U/L (57.1–679.3) compared with 415 U/L (90–1296) in the total AP group (*p* = 0.3017).

The patient characteristics and the comparisons between the overall AP cohort and HTG-AP patients are listed in table below (Table 3).

**Table 3:** Patient characteristics with comparisons between the overall AP cohort and HTG–AP patients

Item	Acute abdominal pain	Acute pancreatitis	HTG–AP	*p*-value
Number of cases	1053	226	5	
Male sex	484 (46)	140 (62)	4 (80)	0.4204
Mean age (years)	53 ± 19	57 ± 16	41 ± 10	**0.0286**
Hospitalization duration (days)	7 (4–14)	8 (6–17)	9 (8–18)	0.534
Intensive care unit admission	63 (6)	16 (7)	1 (20)	0.2738
Obesity	390 (37)	95 (42)	4 (80)	0.0897
Uncontrolled diabetes	74 (7)	36 (16)	3(60)	**0.0093**
History of diabetes	126 (12)	38 (17)	4 (80)	**0.0003**
HbA1c (%)	5.4 (5.2–6.3)	5.7 (5.5–7.2)	8.8 (6.7–10.2)	**0.0304**
History of AP	32 (3)	36 (16)	1 (20)	0.8061
Death during admission	10 (1)	5 (2)	0	0.7367
Triglycerides (mg/dL)	118 (81–158)	116 (83–193)	3002 (1118–4113)	** < 0.0001**
Total cholesterol (mg/dL)	149 (127–188)	154 (136–196)	438 (196–756)	**0.0046**
Maximal lipase (U/L)	42 (34–123)	415 (90–1296)	101.3 (57.1–679.3)	0.3017
Maximal amylase (U/L)	35 (23–78)	163 (40–492)	64 (29–316)	0.463
Maximal CRP (mg/dL)	2.3 (0.9–4.4)	7.8 (6.3–15.4)	6.9 (4.7–17.7)	0.632
Leucocytes (/µL)	10.2 (7.8–12.3)	14.3 (12.2–16.7)	13.3 (10–19.75)	0.645
Maximal GPT (U/L)	29 (20–44)	42 (32–61)	51 (36–65)	0.6781
Maximal GOT (U/L)	24 (19–38)	40 (31–58)	42 (33–70)	0.7012
Maximal bilirubin (mg/dL)	0.65 (0.43–0.96)	0.89 (0.74–1.5)	0.92 (0.76–1.2)	0.845
Maximal creatinine (mg/dL)	0.84 (0.74–1.43)	1.2 (0.86–1.85)	1.1 (0.91–1.2)	0.543
Maximal ionized calcium (mmol/L)	1.24 (1.12–1.29)	1.26 (1.16–1.31)	1.28 (1.12–1.32)	0.782
Minimal ionized calcium (mmol/L)	1.18 (1.11–1.24)	1.11 (1.02–1.16)	1.13 (1.07–1.17)	0.791

Continuous variables are presented as mean ± standard deviation (SD) or as median with interquartile range (Q1–Q3), as appropriate. Categorical variables are expressed as absolute numbers (*n*) and percentages (%). *p*-values comparing the overall AP cohort and HTG–AP patients were calculated using Student’s *t*-test, *χ*^2^ test, or the Mann–Whitney *U* test, as appropriate. Significant results (*p* < 0.05) are in bold

### Determination and Response to Elevated Inpatient Triglyceride Levels in a Second Cohort

We analyzed the awareness of TG determination in clinical routine using an alternative approach. To this end, we extracted laboratory analysis data corresponding to TG levels above 500 mg/dL, indicative of possible moderate to severe HTG, and subsequently examined the specific patient records for evidence of a direct, deliberate response to these determinations. We identified 237 matching patient files. Of these, 67.5% were male and 32.5% were female, with an average age of 55 years ± 15. The patients were treated by different medical specialists for various reasons. The following tables provide all relevant information regarding this patient cohort (Tables 4, 5, 6). The median TG level was 742 mg/dL (665–920). Of the 237 patients, 62 (26.16%) were diagnosed with HTG. Of these patients, 27 (11.3%) received a first-time diagnosis, while 35 (14.7%) had previously been diagnosed with HTG. Interestingly, 175 patients (73.8%) were not diagnosed with or suspected of having HTG. 101 Patients (42.6%) had received triglyceride-lowering therapy. Of these, therapy was initiated for hypercholesterolemia without considering HTG in 39 patients (16.4%). Of those receiving therapy, 62 (61.3%) received statin monotherapy, while 26 (25.7%) received a combination of several possible forms of therapy. Of the 62 patients diagnosed with HTG, 17 were referred for HTG-targeted follow-up. Of these patients, 10 were referred to a specialist lipid clinic and 7 to an endocrinologist. Mixed hyperlipidemia was present in 48.5% of cases, and the etiology of HTG was not determined in 217 (91.5%) cases.

**Table 4:** Etiology of hypertriglyceridemia (HTG)

HTG-etiology	*n* (%)
Familial hypertriglyceridemia	5 (2.11)
Hyperchylomicronemia	4 (1.69)
Secondary hypertriglyceridemia	11 (4.64)
Not further defined	217 (91.56)

**Table 5:** Reasons for presentation in HTG cases

Reason	*n* (%)
Follow-up or complications after kidney transplant under immunosuppression	46 (19.41)
Sepsis	14 (5.91)
Lymphoma	14 (5.91)
Acute lymphoblastic leukaemia	7 (2.95)
Acute myeloid leukemia	5 (2.11)
COVID pneumonia	13 (5.49)
Hyperglycemic derailment	6 (2.53)
Others	132 (55.70)

**Table 6:** Demographic and clinical characteristics of patients with HTG

Item	Patients with HTG (> 500 mg/dL)
Number of cases	237
Male sex	160 (67.5)
Mean age (years)	55 ± 15
Hospitalization duration (days)	6 (4–13)
Intensive care unit admission	19 (8)
Obesity	80 (34)
Uncontrolled diabetes	88 (37)
History of diabetes	130 (55)
HbA1c (%)	7 (6.2–8.4)
History of AP	32 (13.5)
Death during admission	7 (3)
Triglycerides (mg/dL)	742 (665–920)
Total cholesterol (mg/dL)	221 (169–258)
Maximal lipase (U/L)	42 (26–77)
Maximal amylase (U/L)	31 (19–58)
Maximal CRP (mg/dL)	0.93 (0.42–8)
Leucocytes (/nL)	8.3 (7.2–12.4)
Maximal GPT (U/L)	44 (32–61)
Maximal GOT (U/L)	39 (28–51)
Maximal bilirubin (mg/dL)	1.1 (0.83–1.54)
Maximal creatinine (mg/dL)	1.3 (0.96–1.73)
Maximal ionized calcium (mmol/L)	− 1.25 (1.13–1.28)
Minimal ionized calcium (mmol/L)	1.13 (1.07–1.121)

Continuous variables are presented as mean ± standard deviation (SD) or as median with interquartile range (Q1–Q3), as appropriate. Categorical variables are expressed as absolute numbers (*n*) and percentages (%)

**Fig. 2:**
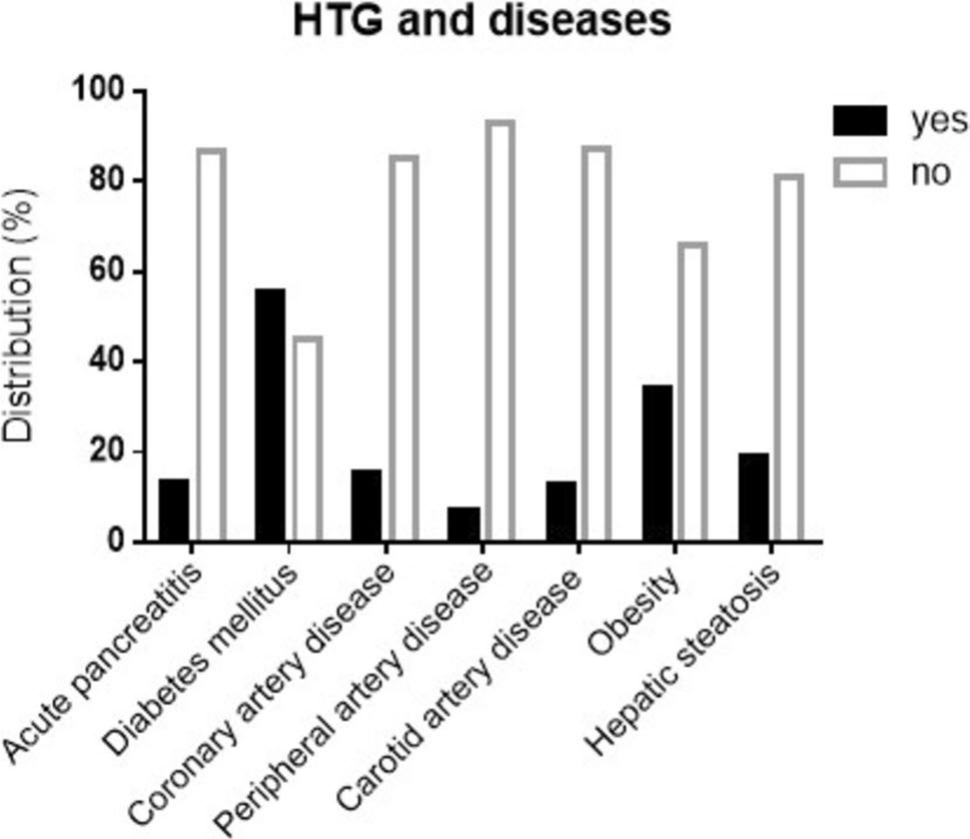
Distribution of comorbidities in patients with HTG

Of all cases, 13.5% had a history of AP, 55% had diabetes mellitus, 15% had coronary heart disease (CHD), 6.9% had peripheral arterial disease (PAD), 12.9% had carotid artery disease (CAD), 34% were obese, and 18.9% had hepatic steatosis (Fig. 2). Interestingly, only 22% of patients with a history of AP were taking TG lowering medication prior to admission.

## Discussion

AP caused by HTG is a dangerous and often even lethal disease that needs to be identified and treated as early as possible [9]. Current guidelines from the “Deutsche Gesellschaft für Gastroenterologie, Verdauungs- und Stoffwechselkrankheiten” (DGVS) and others prioritize the early detection of TG levels in cases of suspected or diagnosed AP, although precise time frames are not provided***.*** However, a study by Dong et al. suggested an early detection within the first 48 h of pancreatitis onset [10], which showed the strongest correlation with disease diagnosis and assessment.

In our monocentric, retrospective analysis, we found that TG measurement, if performed at all, was often delayed beyond this time frame. Further, we provide—to our knowledge for the first time—real world data on timing and time delay in the determination of TG in AP beyond trial design, by that addressing a major practical problem in everyday practice.

The overall rate of TG measurement during hospitalization among patients with AP was 55%. Although relatively low, this rate exceeded the 28% reported in a Canadian study investigating HTG–AP [11].

The low rates of TG determination, especially on admission (10.2% for acute abdominal pain, 12.8% for AP), suggest a systematic failure in the early detection of HTG. This may indicate a lack of awareness among clinical decision makers to consider HTG as a possible cause. In addition, the identification is significantly influenced by the presence of different disciplines in the emergency department, which set different priorities for the initial determination of laboratory parameters. Early measurement of serum TG levels can provide valuable prognostic information for patients who are severely or critically ill. A large ICU cohort study of patients with sepsis demonstrated a U-shaped association between TG levels and 30-day mortality, with both high and markedly reduced concentrations being linked to worse outcomes [12]. In a study of patients with acute liver failure by Manka et al., low TG levels reflected advanced hepatic dysfunction and independently predicted mortality [13]. These studies highlight that a pronounced decline in TG levels from normal initial levels indicates severe metabolic dysregulation and impending deterioration. Therefore, prompt TG assessment at admission and during follow-up, when appropriate, can aid the early identification of high-risk patients and support the timely intensification of monitoring and therapeutic strategies.

In HTG pancreatitis, initial TG values are sometimes relatively low due to the severe symptoms that lead to fasting induced by pain before patients are admitted to A&E [14]. It is therefore necessary to optimize diagnostic algorithms in the disciplines that are most frequently confronted with such cases.

The median time taken to determine TG (24 h for abdominal pain and 48 h for AP) is problematic. A delay in TG determination, particularly in high-risk patients (e.g. those with diabetes or obesity), often means that the cause remains unidentified and the disease progresses without appropriate therapy. HTG is the third most common trigger, yet it was identified as the cause in only 5 of 226 cases, while no cause was identified in 66 cases. This may be due to the non-specific presentation and lack of TG measurement on admission. Delayed determination of the TG level can lead to false-negative results. The rapid drop reduces the diagnosis rate of HTG-AP. It is therefore crucial to establish diagnostic strategies and determine the current TG level as quickly as possible in cases of unclear abdominal pain or AP. Modest increases in baseline TG levels (measured during the first outpatient visit after the initial hospitalization for AP) were found to be linked to a higher risk of AP [15]. Only 16% of patients with HTG and acute abdominal pain and 35.5% of patients with HTG and pancreatitis received TG-lowering therapies. For this reason, the establishment of in-hospital treatment protocols and the stronger embedding of HTG management in clinical guidelines is essential.

A recent cohort study involving 1233 patients in China demonstrated a significant and progressive increase in mortality, as well as other adverse clinical outcomes such as organ failure and pancreatic necrosis, in association with higher levels of HTG [16]. Our analysis revealed high rates of comorbidities, including diabetes mellitus (55%), obesity (34%), and cardiovascular disease (e.g. CHD 15%, CAD 12.9%). It also revealed that the underlying cause of HTG was unidentified in 91.5% of cases, highlighting the importance of identifying the cause for ensuring appropriate treatment. Consistent HTG management helps to avoid long-term consequences.

There is currently a lack of meaningful data on the long-term outcomes for patients with untreated HTG. Regular lipid profile assessments during check-ups and increased awareness and training among healthcare providers are essential for the early detection of dyslipidemia. Combining these measures with early, targeted treatment for at-risk patients is crucial for improving prognosis and minimizing complications.

Our study has several limitations. Firstly, the retrospective design restricts the generalizability of the findings and precludes the evaluation of long-term management strategies and clinical outcomes following hospitalization. Secondly, the relatively small sample size and inclusion of patients from a single center may introduce selection bias and limit applicability to a broader population. Thirdly, precisely determining the etiology of AP was challenging in some cases due to the coexistence of multiple potential causative factors. For example, although alcohol consumption is independently associated with an increased risk of AP, it can also contribute to elevated serum TG levels, which makes identifying the primary cause more difficult.

## Conclusions

In conclusion, this study presents the first data on the timing of TG measurements in patients with AP. It reveals a significant delay: only 12.8% of patients were tested on admission, resulting in an overall measurement rate of just 55%. Given the rapid drop in TG levels, delayed measurement can lead to underdiagnosis. The low rate of TG-lowering therapy further highlights the need for standardized protocols. Consequently, efforts to improve early detection and treatment in this field are warranted.

## Supplementary Information

Below is the link to the electronic supplementary material.

(DOCX 15 kb)

## Data Availability

No datasets were generated or analysed during the current study.
