# Supplementary material for: Hypertriglyceridemia Screening in Acute Pancreatitis: Diagnostic Blind Spot in Clinical Routine
Source: Dig Dis Sci. 2025 Sep 13;71(2):709–15. doi: 10.1007/s10620-025-09366-4 (PMC12946338; doi:10.1007/s10620-025-09366-4)
Supplement: Supplementary file 1 — (DOCX 15 kb) [file 10620_2025_9366_MOESM1_ESM.docx]

***Specialty n (%)***

General surgery 73 (28.85)

Neurosurgery 23 (9.09)

Trauma surgery 5 (1.98)

Oral and maxillofacial surgery 2 (0.79)

Surgical intensive care unit 24 (9.49)

Occupational medicine 1 (0.40)

Neurology 36 (14.23)

Hematology 32 (12.65)

Gastroeneterology 23 (9.09)

Cardiology 16 (6.32)

Infectious diseases 1 (0.40)

Intensive care unit 4 (1.58)

General internal medicine 13 (5.14)

Table 1. Medical disciplines in which patients with hypertriglyceridemia (HTG) are treated.
